# Right Limbic FDG-PET Hypometabolism Correlates with Emotion Recognition and Attribution in Probable Behavioral Variant of Frontotemporal Dementia Patients

**DOI:** 10.1371/journal.pone.0141672

**Published:** 2015-10-29

**Authors:** Chiara Cerami, Alessandra Dodich, Sandro Iannaccone, Alessandra Marcone, Giada Lettieri, Chiara Crespi, Luigi Gianolli, Stefano F. Cappa, Daniela Perani

**Affiliations:** 1 Vita-Salute San Raffaele University, Milan, Italy; 2 Division of Neuroscience, San Raffaele Scientific Institute, Milan, Italy; 3 Clinical Neuroscience Department, San Raffaele Hospital, Milan, Italy; 4 Nuclear Medicine Department, San Raffaele Hospital, Milan, Italy; 5 NeTS Center, Istituto Universitario di Studi Superiori, Pavia, Italy; University G. D'Annunzio, ITALY

## Abstract

The behavioural variant of frontotemporal dementia (bvFTD) is a rare disease mainly affecting the social brain. FDG-PET fronto-temporal hypometabolism is a supportive feature for the diagnosis. It may also provide specific functional metabolic signatures for altered socio-emotional processing. In this study, we evaluated the emotion recognition and attribution deficits and FDG-PET cerebral metabolic patterns at the group and individual levels in a sample of sporadic bvFTD patients, exploring the cognitive-functional correlations. Seventeen probable mild bvFTD patients (10 male and 7 female; age 67.8±9.9) were administered standardized and validated version of social cognition tasks assessing the recognition of basic emotions and the attribution of emotions and intentions (i.e., Ekman 60-Faces test-Ek60F and Story-based Empathy task-SET). FDG-PET was analysed using an optimized voxel-based SPM method at the single-subject and group levels. Severe deficits of emotion recognition and processing characterized the bvFTD condition. At the group level, metabolic dysfunction in the right amygdala, temporal pole, and middle cingulate cortex was highly correlated to the emotional recognition and attribution performances. At the single-subject level, however, heterogeneous impairments of social cognition tasks emerged, and different metabolic patterns, involving limbic structures and prefrontal cortices, were also observed. The derangement of a right limbic network is associated with altered socio-emotional processing in bvFTD patients, but different hypometabolic FDG-PET patterns and heterogeneous performances on social tasks at an individual level exist.

## Introduction

The behavioral variant of frontotemporal dementia (bvFTD) is a disorder of social brain. Changes of personality and social conduct are main features of the disease since its onset [1–2]. Mild disorders of personal conduct and socially inappropriate behaviors may result from a failure to correctly identify, interpret or react to socio-emotional signals coming from the environment, possibly associated with potential reward or punishment value. These changes progressively erode patient’s social, professional, and familial dimensions leading to a dramatic modification of patient and relatives relationship network.

In the last decade, structural and functional imaging have shown that many of the cognitive sub-processes most likely involved in socio-emotional processing, such as emotion recognition, empathy and affective theory of mind, are mediated by specific fronto-limbic structures (e.g., orbitofrontal cortex, anterior/middle cingulate cortex, amygdala, insula, and temporal pole) [3–5]. The so-called “salience network” is indeed the main large-scale functional network targeted by the neurodegeneration in this pathological condition [6]. It comprises the anterior cingulate and fronto-insular cortices, in close interaction with other limbic and subcortical structures. The derangement of this network, together with the reduced connectivity of fronto-limbic white-matter tracts, can have an effect on patient’s response to social stimuli, affecting the sensitivity to the negative consequences of the social acts [7].

As shown by several experimental paradigms assessing single and multiple facets of social cognition, bvFTD patients are impaired in recognition of basic and complex emotions, social decision-making, comprehension and inference of other’s mental states, maintaining awareness of own social behaviour [8–20]. Severe deficits in facial emotion recognition particularly for negative and complex emotions are frequently observed (see [8] for a review). In addition, according to the multifactorial nature of socio-emotional processing, reduced emotion recognition is often associated with disruption of other aspects of social cognition. Deficits in high-order social information processing are more frequently reported in bvFTD compared to Alzheimer’s disease (AD) [9–10]. The presence of severe social cognition disorders (e.g., ToM deficits or loss of empathy) in bvFTD is also strongly supported by an ample research literature in the last decade. Many papers directly compared performances in bvFTD to those of AD patients on different socio-emotional processing tasks and concluded for a more widespread and severer impairment in bvFTD (see as examples [15–20]). This certainly does not mean that such cognitive functions are spared in AD or other neurodegenerative conditions (e.g., dementia with Lewy bodies, corticobasal degeneration or progressive supranuclear palsy) (see as examples [21–23]). However, as also supported by two recent meta-analyses [9–10], controlling for general cognition, impairments of high-order social information processing are significantly more frequent in bvFTD than in other dementias. Performances on empathy tasks are particularly impaired and may not be merely attributed to difficulties in other cognitive domains, as supported by Baez and colleagues’ findings [14] showing that empathic concern (i.e., "other-oriented" feelings of sympathy and concern for unfortunate others) is not predicted by executive functioning scores.

The above mentioned literature strongly supported the inclusion of “early empathy/sympathy loss” within the core clinical criteria for diagnosis of bvFTD [24]. In further support of the bvFTD diagnosis, there is the neuroimaging evidence of MRI atrophy and FDG-PET hypometabolism in frontal lobes and in the anterior temporal regions [24]. This metabolic pattern is, however, limited since reduction of glucose metabolism can involve other brain regions within the fronto-limbic system, the temporal lobes, as well as the basal ganglia. Previous FDG-PET studies have shown hypometabolism in the ventromedial frontopolar cortex, reported as a functional hallmark of the early bvFTD phase [25], as well as in the anterior cingulate cortex, the anterior insula, the temporal poles, and the ventral basal ganglia, caudate and thalamus [26–30]. All these can be considered metabolic signatures of bvFTD.

Several studies explored the MRI brain structural correlates of social cognition impairments in bvFTD [12–13, 15, 31–34] Among the few investigations with FDG-PET metabolic changes [35–37], Zahn and colleagues [35], using a semantic discrimination task for social and animal function knowledge, provided evidence in a large series of frontotemporal lobar degeneration cases for conceptual social knowledge impairments that correlated with an anterior temporal dysfunction. Le Bouc et al. [36] reported different impairments in bvFTD and AD for two aspects of ToM (belief inference and self-perspective inhibition). Compared to AD, bvFTD showed a specific deficit of the self-perspective inhibition that was related to a reduced glucose metabolism in the right prefrontal cortex. Moll and colleagues [37] showed reduced prosocial sentiments associated with hypometabolism in frontopolar cortex and septal areas.

None of these studies explored the processing of social signs with affective cues, whose impairment is a fundamental feature of bvFTD [8]. Here, we report the FDG-PET metabolic correlates of impaired emotion recognition and attribution in bvFTD patients. On the basis of the evidence coming from behavioral and imaging studies, we predicted that impaired performances on basic emotion recognition and attribution would be correlated with metabolic dysfunctions in the fronto-limbic structures.

## Materials and Methods

### Participants

Seventeen mild dementia patients (10 male and 7 female; age 67.88±9.92; education 11.94±3.90; Mini-Mental State Examination (MMSE) raw score 23.6±3.83; CDR global score 0.5–1) fulfilling clinical criteria for probable bvFTD [24] were included in the study (Table 1). All patients were consecutively recruited from the Department of Clinical Neurosciences, San Raffaele Scientific Institute (Milan, Italy), and evaluated by a team of experienced behavioral neurologists and neuropsychologists. All subjects underwent full neurological examination, neurobehavioral and cognitive assessment. Both patients and caregivers underwent a structured clinical interview. Patients were preliminary screened for the main genes causative for autosomic dominant frontotemporal dementia (i.e., *GRN*, *MAPT* and *C9orf72*). None of patients carried known mutations. Brain MRI/CT and FDG-PET data were collected for diagnostic purposes to support the diagnosis. As required for the diagnosis of probable bvFTD [24], frontal and/or anterior temporal atrophy on MRI/CT or hypometabolism on FDG-PET was found in each single case. FDG-PET was more informative as supportive biomarker than structural imaging, showing indeed brain metabolic changes even in those cases with no alteration on structural imaging (n = 9/17). Structural imaging data showed mild frontal and anterior temporal atrophy only in the 47% of the sample, the remaining showed no relevant changes on MRI. No clear asymmetric atrophy pattern was detected on structural brain imaging. In addition, structural imaging information was acquired in each case in order to exclude the presence of white matter hyperintensities and lacunes of presumed vascular origin. CSF β-Amyloid, Tau and p-Tau values were available in 7/17 patients. In no case, β-Amyloid and Tau changes typical of AD were found.

**Table 1:** Clinical and demographical features of patient sample are shown in the upper part of the table. Below that, brain areas presenting right, left or bilateral FDG-PET hypometabolism at the voxel-based SPM single-subject analysis are shown. *M*: male; *F*: female; *MMSE*: Mini Mental State Examination raw score; *L* and *R*: left and right hemisphere.

	#1		#2		#3		#4		#5		#6		#7		#8		#9		#10		#11		#12		#13		#14		#15		#16		#17	
*Gender*	M		F		M		F		F		F		M		M		M		M		F		F		M		M		M		M		F	
*Age in years*	64		83		55		65		49		69		50		78		65		63		68		82		75		71		77		78		67	
*Education in years*	18		13		13		11		17		8		8		5		5		6		13		13		12		17		11		16		13	
*Disease duration*	12		36		24		60		22		18		48		24		24		24		24		112		118		17		12		39		24	
*MMSE raw score*	30		24		23		22		11		19		25		24		22		27		25		21		24		25		25		14		30	
	L	R	L	R	L	R	L	R	L	R	L	R	L	R	L	R	L	R	L	R	L	R	L	R	L	R	L	R	L	R	L	R	L	R
*Dorsolateral Prefrontal Cortex*	x	x	x	x	x	x	x	x	x	x	x	x	x	x		x		x	x															
*Inferior Frontal Gyrus*		x	x	x	x	x	x	x	x	x	x	x	x	x			x	x	x	x	x	x	x	x	x	x	x	x	x	x	x	x		
*Superior Temporal Gyrus*							x				x		x	x	x	x		x	x	x		x	x	x	x	x					x		x	
*Ventromedial Frontopolar Cortex*	x	x		x	x	x	x	x	x	x	x	x	x	x	x	x	x	x	x	x	x	x	x	x	x	x								
*Orbitofrontal Cortex*			x	x	x	x	x	x	x	x	x	x	x	x			x	x	x	x	x	x	x	x	x	x	x	x	x	x				
*Anterior and Middle Cingulate Cortex*	x	x	x	x	x	x			x	x	x	x	x	x	x	x	x	x	x	x	x	x	x	x	x	x	x	x	x	x	x	x		
*Temporal Pole*			x	x	x		x	x	x	x	x	x	x	x	x	x	x	x	x	x	x		x	x	x	x		x	xx	x	x	x	x	
*Anterior Insula*		x	x	x	x	x	x	x	x	x	x	x	x	x	x	x	x	x	x	x	x	x	x	x	x	x	x	x	x	x	x	x	x	x
*Amygdala*				x					x	x	x	x			x	x	x	x	x	x		x	x	xx	x	x	x	x	x	x	x	x	x	
*Hippocampal structures*							x	x	x		x				x	x	x	x	x			x	x	x	x	x	x	x	x	x	x		x	
*Nucleus Accumbens*							x	x		x	x	x	x	x		x	x	x	x	x	x	x	x	x	x	x			x	x	x		x	
*Caudate*	x	x					x		x	x	x	x	x	x	x	x	x		x	x	x		x	x	x	x			x	x	x	x		
*Thalamus*							x	x			x		x	x					x			x			x	x	x	x						

All subjects, or their informants/caregivers, gave written informed consent to the experimental procedure that had been approved by the Ethical Committee of San Raffaele Hospital.

### Emotion recognition and attribution assessment

Standard neuropsychological battery included measures of global cognitive functioning (i.e., Mini Mental State Examination), memory (i.e., Digit Span Forward, immediate and delayed recall of Rey Auditory Verbal Learning, recall of copy of the Rey-Osterrieth figure) and executive functions (i.e., Raven Coloured Progressive Matrices; Digit Span backward; letter (P-F-L) and category (animals-fruits-car brands) fluency tests; Cognitive Estimation Task; Stroop Interference Test or Wisconsin Card Sorting Test), language abilities (i.e., Token test, picture naming and single word comprehension subtests of CAGI battery), visuo-spatial abilities (i.e., copy of the Rey-Osterrieth figure) and behavioral disturbances (i.e., Frontal Behavioral Inventory, Neuropsychiatric Inventory and Frontal Assessment Battery).

A second-level socio-emotional battery assessing the recognition of basic emotions from facial expression (Ekman 60-faces task–EK-60F) and the attribution of mental states to other individuals (Story-based Empathy Task–SET) was administered to each patient. The EK-60F is a well-known task of basic emotion recognition that consists of 60 b/w pictures from the Ekman and Friesen series of Picture of Facial Affect, which depict the faces of 10 actors, each displaying six basic emotions (i.e., happiness, sadness, anger, fear, surprise, disgust). Ek-60F patients’ performances were evaluated according to the Italian norms [38]. The SET task is a non-verbal task, developed in our laboratory with original cartoons and standardized for the Italian population [39], which assessed attribution of mental states to other individuals requiring the recognition of their intentions (i.e., SET-IA) versus emotional states (i.e., SET-EA), as well as the ability to infer physical causal relationships (i.e., SET-CI) devoid of social components (see [12,39] for further details on SET task). Normative scores for the Italian population [38–39] were used to evaluate patients’ performances.

In order to investigate patients’ empathic attitude and to correlate it with the socio-emotional performances of the patients, the Interpersonal Reactivity Index–IRI [15] was administered to caregivers. It is a 28-item questionnaire that includes four 7-item subscales assessing different aspects of empathy previously used in dementia patients [15]. Caregivers were asked to rate how well each of 28 statements reflected the current behaviour of the participant on a scale of 1 (does not describe at all) to 5 (describes very well). The Fantasy (“*When I am reading an interesting story or a novel*, *I imagine how I would feel if the events in the story were happening to me*”) and the Perspective-Taking (“*I sometimes try to understand my friends better by imagining how things look from their perspective*”) subscales measure cognitive empathy, while emotional empathy is assessed through the Empathic Concern (“*I often have tender*, *concerned feelings for people less fortunate than me*”) and the Personal Distress subscales (“*Being in a tense emotional situation scares me*”). Due to the lack of normative scores, mean and standard values reported in literature [15] were used to evaluate our sample’s performances.

### FDG-PET imaging

#### FDG-PET scan acquisition

FDG-PET acquisitions were performed at the Nuclear Medicine Unit, San Raffaele Hospital (Milan, Italy). Before radiopharmaceutical injection of FDG (185–250 Mbq: usually, 5–8 mCi via a venous cannula), subjects were fasted for at least 6 hours and their blood glucose level was <120 mg/dL. All images were acquired with a Discovery STE (GE Medical Systems, Milwaukee, WI) multi-ring PET tomography (PET-CT) system (time interval between injection and scan start = 45 minutes; scan duration = 15 minutes). Images were reconstructed using an ordered subset expectation maximization (OSEM) algorithm. Each PET phase was corrected for attenuation with CT data of the corresponding phase. For each PET scan has been acquired 47 transaxial tomographic slices of 4.25 mm, re-oriented into the coronal and the sagittal planes. The emission images were then reconstructed using a filtered back-projection, using the software provided by the manufacturers.

All subjects gave written informed consent, following detailed explanation of the FDG-PET procedure.

#### FDG-PET data pre-processing and statistical analysis

Image processing and statistical analysis were performed according to validated procedures [40–41]. In particular, normalization procedure was performed at the individual level to a dementia-specific Statistical Parametrical Mapping (SPM) FDG-PET template [40]. Each patient scan was then tested for relative “hypometabolism” on the basis of a validated procedure that includes comparison with a large normal image database of FDG-PET on a voxel-by-voxel basis [41]. Age was included as a covariate. Proportional scaling was used to remove inter-subject global variation in PET intensities. The threshold was set at p = 0.05, FWE-corrected for multiple comparisons at the voxel level. Only clusters containing more than 100 voxels were deemed to be significant.

In order to evaluate the regional commonalities in the pattern of FDG-PET hypometabolism in the bvFTD patients performing at or below the 5^th^ percentile in the tasks assessing processing of affective cues (i.e., Ek-60F global score and SET-EA sub-score) (n = 7; Table 2), we computed a whole-brain group analysis (i.e., one-sample t-test) using the contrast images resulting from each first-order “single-subject” analysis. The p-value (uncorrected) was set at p<0.001.

**Table 2:** Performances of bvFTD patients on socio-emotional tasks are shown as adjusted scores according to the Italian normative data for both the Story-based Empathy Task (SET) and the Ek-60F (Ekman 60-Faces Task), and as raw scores for the Interpersonal Reactivity Index (IRI). Equivalent scores = 0 (performance under the 5° percentile according to the Italian normative data) are shown in bold, while equivalent scores = 1 (performance between the 5° and the 10° percentile according to the Italian normative data) in italics. For IRI scores, values reported as cut-off are obtained using as reference score reported in [14]. *SET GS*: SET global score; *SET-IA*: SET Intention Attribution sub-scale score; *SET-EA*: SET Emotion Attribution sub-scale score; *SET-CI*: SET Causal Inferences sub-scale score; *EK-60F GS*: Ek60-F global score; *ERA*: emotion recognition and attribution index; *IRI-PT*: IRI Perspective Taking; *IRI-F*: IRI Fantasy; *IRI-EC*: IRI Empathic Concern; *IRI-PD*: IRI Personal Distress. *N*.*E*. = not evaluable for perseveration (# 16) or attention and/or working-memory disorders (# 4, 5, 6, 9). *N*.*A*. = not available.

	#1	#2	#3	#4	#5	#6	#7	#8	#9	#10	#11	#12	#13	#14	#15	#16	#17	All sample	Cut-off
*SET GS*	11.91	*10*.*14*	16.01	N.E.	*10*.*45*	N.E.	12.21	**7.52**	N.E.	16.27	**4.05**	**8.14**	11.08	*8*.*94*	*9*.*11*	N.E.	**7.05**	10.2±3.6	8.29
*SET-IA*	*2*.*99*	4.08	6	N.E.	5.10	N.E.	5.15	*2*.*35*	N.E.	6	**1.05**	4.08	5.05	**0**	4.03	N.E.	**2.05**	3.6±1.9	2.34
*SET-EA*	4.95	4.12	5.01	N.E.	*2*.*91*	N.E.	*3*.*13*	**1.36**	N.E.	4.18	**0.05**	**1.12**	**1.7**	*2*.*98*	**2.09**	N.E.	*3*.*05*	2.8±1.6	2.20
*SET-CI*	3.9	**2.13**	5.01	N.E.	*2*.*86*	N.E.	4.21	4.5	N.E.	6	*3*.*05*	*3*.*13*	5.07	6	*3*.*1*	N.E.	**2.05**	4±1.9	2.41
*Ek-60F GS*	54	**34.36**	**33.98**	**33.5**	N.E.	N.E.	**36.02**	**27.93**	**25.07**	43.88	**20.45**	**36.35**	**34.84**	**25.24**	**35.74**	**28.15**	51.5	36.2±9.8	37.46
*Ek-60F Surprise*	9	**4**	8	8	N.E.	N.E.	7	**3**	**2**	7	**2**	*6*	*6*	**4**	*6*	**3**	7	5.7±2.1	6
*Ek-60F Happiness*	10	**8**	10	**8**	N.E.	N.E.	*9*	**7**	**8**	10	**0**	*9*	**8**	**7**	*9*	**7**	10	8.1±2.8	9
*Ek-60F Fear*	8	3	**0**	3	N.E.	N.E.	*2*	*2*	3	3	5	5	**1**	**0**	*2*	4	7	3.2±2.6	2
*Ek-60F Disgust*	10	7	*4*	**3**	N.E.	N.E.	5	**2**	**1**	6	5	*4*	7	9	**1**	**2**	9	5.7±2.8	4
*Ek-60F Anger*	8	**4**	*5*	*5*	N.E.	N.E.	**3**	**3**	**2**	6	**3**	**2**	*5*	**0**	7	6	7	4.4±2.3	5
*Ek-60F Sadness*	8	**3**	6	*4*	N.E.	N.E.	8	**2**	**3**	7	**2**	5	*4*	**3**	5	**2**	9	5.2±2.4	4
*IRI-PT*	N.A.	18	13	7	24	29	14	11	N.A.	14	17	14	9	15	32	20	19	16±6	16.4±7.6
*IRI-F*	N.A.	14	35	12	28	21	14	12	N.A.	16	13	17	7	15	16	29	14	15.7±6.9	14.8±6.2
*IRI-EC*	N.A.	23	23	11	20	19	15	15	N.A.	29	20	17	9	28	12	10	27	19.8±6.7	22.6±7.9
*IRI-PD*	N.A.	18	25	13	26	29	30	16	N.A.	21	23	17	15	18	21	21	28	21.1±4.9	21.6±6.7
*ERA score*	103.8	75.56	84.08	-	-	-	67.32	41.53	-	85.68	20.95	47.55	45.54	55.04	56.64	-	82	63.8±23.4	-

#### Correlation analyses

With the aim to identify in bvFTD patients brain regions in which reduced glucose metabolism was correlated with low performance in the social tasks, we extracted for each patient the value of relative hypometabolism from the contrast image in the single-subject analysis (i.e., each patient vs. normal database). We selected the regions of interest (ROIs) found hypometabolic in single cases SPM resulting maps (see Table 1), and, namely, the dorsolateral prefrontal cortex, the ventromedial frontopolar cortex, the orbitofrontal cortex, the inferior frontal gyrus, the anterior and middle cingulate cortex, the insula, the amygdala, the temporal pole, the superior temporal gyrus. These regions are also known to be the neural correlates of different aspects of emotion recognition and attribution [3–5, 42].

Then, we computed correlations between the extracted values of hypometabolism and the measures of social functioning. According to the main aim of this study, we calculated an index of emotion recognition and attribution combining the Ek-60F adjusted global score and the SET-EA sub-scale adjusted score (i.e., ERA index = (SET-EAx10) + Ek-60F global score). These results were corrected for multiple comparisons (FDR).

In addition, both Ek-60F and SET adjusted global and single sub-scale scores were included in the correlation analyses. Due to the non-normal distribution of neuropsychological data and considering the small number of patients, non-parametric statistics were used for correlation analyses between behavioural performance and metabolic metrics (i.e., Spearman r index).

## Results

### Emotion recognition and attribution impairments

First, we analysed patients’ performances on Ek-60F and SET tests according to Italian normative data [38–39]. Of the overall sample, five patients could not perform both Ek-60F and SET tasks due to the presence of working memory deficits and perseverative behavior (Table 2).

SET adjusted global scores resulted impaired (Equivalent score-ES = 0) in the 30% and reduced (ES = 1) in the 23% of the remaining bvFTD sample. SET-EA was the most impaired sub-condition of the task (i.e., ES = 0 in the 38% of patients and ES = 1 in the 31% of patients) (Table 2).

The performance at the Ek-60F recognition task was impaired in the majority of patients (80%). Recognition of both negative and positive emotions was impaired in the sample (Table 2). The analysis of single emotions revealed performances under the cut-off score in the recognition of each emotion in 50% of patients. Recognition of happiness was the most impaired (i.e., 67% of the sample), while fear and disgust were less affected (i.e., 47% impaired).

Performances at the EK-60F and SET tasks did not correlate with disease duration or severity.

The IRI questionnaire showed results comparable with the literature [15] (Table 2). Both IRI global score and the IRI emotional empathy subscales (i.e., Empathic Concern and Personal Distress) were positively correlated with the direct measure of emotion recognition and attribution, namely the ERA index (Spearman r = 0.73, p = 0.01; r = 0.72, p = 0.01).

### FDG-PET SPM metabolic patterns

At the group level, the voxel-based SPM analysis of FDG-PET images revealed a significant pattern of reduced glucose metabolism in the fronto-limbic regions and the temporal lobes supporting the clinical diagnosis of bvFTD [24].

Heterogeneous patterns of brain hypometabolism emerged at the single-subject level analysis. In particular, extensive involvement of limbic structures was present in all cases, while hypometabolism in the dorsolateral prefrontal cortex only in 10 out of 17 patients. There was a significant reduction of glucose metabolism in the ventromedial frontopolar and/or the orbitofrontal cortices (15/17), the anterior/middle cingulate cortex (15/17), the amygdala (13/17), the hippocampal structures (13/17), the temporal pole (16/17) and the insula (16/17). We found also reduced glucose metabolism in the inferior frontal gyrus (15/17) and the superior temporal gyrus (11/17). Four out of nine patients (i.e., patient #4, #6, #16 and #17) with FDG-PET hypometabolism in the left superior temporal gyrus showed also impaired performances at the naming and the verbal fluency tasks. The remaining five (#7, #8, #10, #12, #13) had normal scores at the language tasks included in the standard battery. Metabolic changes spread also to the ventral basal ganglia (i.e., nucleus accumbens, caudate) and the thalamus in all patients except two (i.e., patient #5 and #6). In addition to the widespread fronto-limbic involvement, no patient showed typical imaging findings of AD (i.e., temporo-parietal brain damage with marked hypometabolism of the posterior cingulate cortex and precuneus, sparing of the primary sensorimotor cortex and relative sparing of the occipital lobes) [43], that can suggest a frontal variant of AD [44]. See Table 1 for details on SPM FDG-PET hypometabolic patterns in each patient.

**Fig 1:**
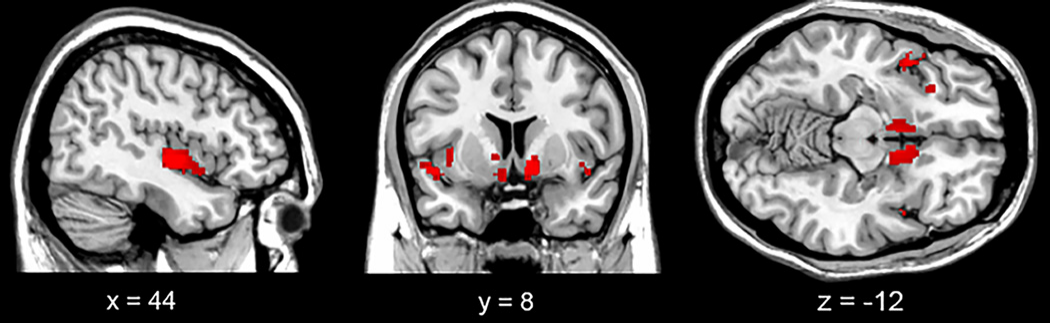
Temporal pole, inferior frontal gyrus, insula and nucleus accumbens FDG-PET hypometabolism in the 7 bvFTD patients impaired on social tasks. Results are overlaid on the SPM structural MNI single-subject template and displayed on sagittal, coronal and axial view. The threshold is settled at p<0.001, minimal cluster extent = 100.

Additionally, FDG-PET SPM group analysis in the seven bvFTD patients impaired on the social tasks revealed a specific hypometabolic pattern limited to the temporal pole and the inferior frontal gyrus, and to the insula and the nucleus accumbens, bilaterally (Fig 1).

### Metabolic correlates of emotion recognition and attribution impairments

Correlation analyses between the brain metabolism and the index of emotion recognition and attribution (i.e., ERA index) were computed only for the 12 patients who performed both SET and Ek-60F tasks (Table 2). There was a negative correlation between the ERA score and the hypometabolism in the right amygdala (Spearman r = -0.82, p = 0.001), the right temporal pole (Spearman r = -0.90, p = 0.0001) and the middle cingulate cortex (Spearman r = -0.61, p = 0.03). With the exception of the last one, all the results survived the FDR correction.

Single task performances on patients who completed the Ek-60F (n = 15) and SET (n = 13) tasks were negatively correlated with hypometabolism in the right amygdala (i.e., Ek-60F global score, Spearman r = -0.57, p = 0.05; SET-EA, Spearman r = -0.83, p = 0.007). SET-EA performances was specifically correlated with the hypometabolism in the right middle cingulate cortex (Spearman r = -0.60, p = 0.04) and right temporal pole (Spearman r = -0.92, p = 0.0001).

**Fig 2:**
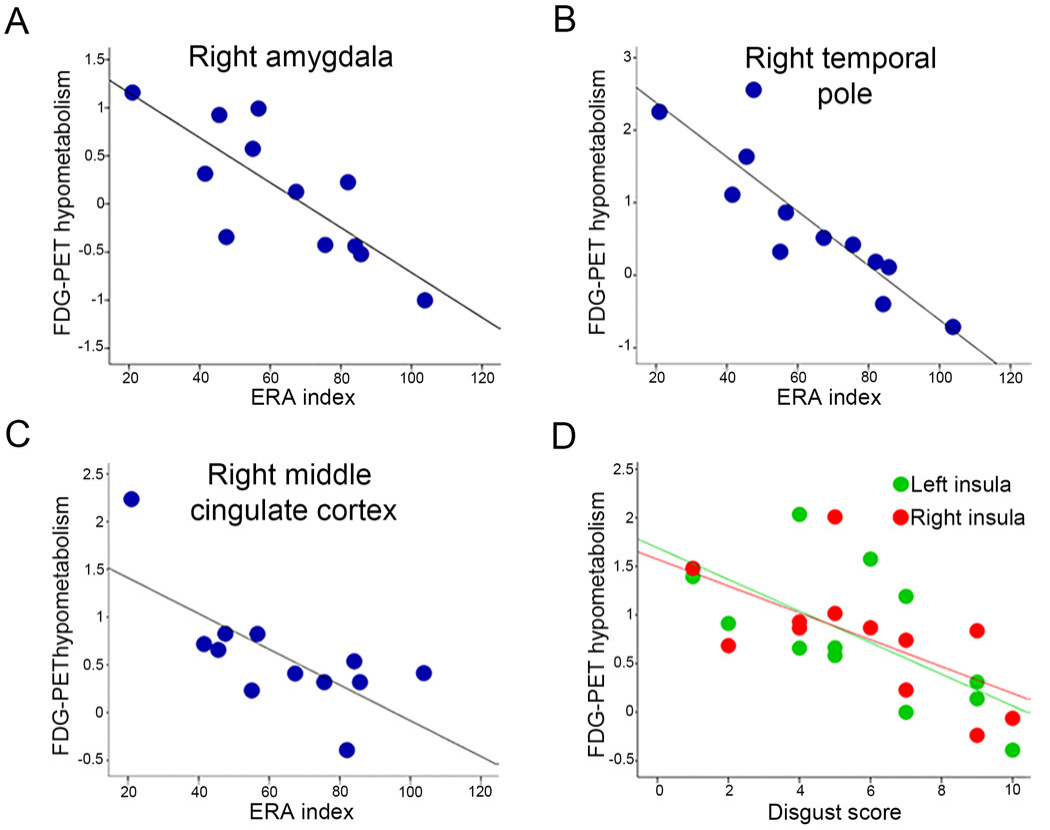
Significant negative correlations between the ERA index and the right amygdala (A), temporal pole (B) and middle cingulate cortex (C) FDG-PET hypometabolism, and between Ek-60F disgust score and the bilateral insula hypometabolism.

Finally, correlation analyses with single emotion Ek-60F sub-scores revealed significant negative correlation between the hypometabolism in the insula, bilaterally, and the disgust recognition score (i.e., right: Spearman r = -0.63, p = 0.02; left: Spearman r = -0.68, p = 0.01). See Fig 2 for correlation analysis results.

## Discussion

Pervasive social and emotional modulation disorders (e.g., violation of social conventions, and emotional blunting) usually dominate the clinical picture of bvFTD from the earliest disease stages [1,2]. A considerable number of researches has recently addressed specific socio-emotional impairments in bvFTD (see for a review [8]). Laboratory-based tasks assessing socio-emotional abilities are particularly important at disease onset, when patients’ performances on executive tasks may be within normal limits [2].

In this study, we used standardized versions of tasks for the assessment of socio-emotional processing (i.e., Ek-60F and SET) and explored in probable bvFTD the performances on recognition of basic emotions and comprehension and inference of other’s mental states, together with the correlations with glucose metabolic dysfunction. We found altered performance on basic emotion recognition tasks in the majority of the bvFTD patients, even though recognition of single emotions was variably impaired among patients (Table 2).

In agreement with previous findings on SET [12], the attribution of emotional states (i.e., SET-EA) was the most impaired sub-condition of the task (Table 2). Emotion processing and empathy abilities are indeed a crucial signature of bvFTD phenotype [8,15]. In line with this, the administration of the IRI questionnaire to caregivers showed reduced scores of cognitive and affective empathy. Though IRI is an indirect measure of empathy, the IRI global score (and particularly the IRI emotional empathy sub-scores) showed consistent positive correlation with the ERA index, the global direct measure of patient’s emotion recognition and attribution ability.

The analyses of metabolic changes in single patients showed variability in the patterns of neuronal dysfunction. Namely, we found that, while fronto-limbic system involvement is a shared feature (Table 1), dorsolateral prefrontal hypometabolism was present in only in subset of bvFTD patients.

The consistent metabolic reductions within the limbic system, including also the subcortical structures, provided crucial *in vivo* evidence for a selective disease-specific vulnerability of the frontal basal-insular-temporal networks in bvFTD patients [7]. The insula was consistently involved in each and every patient.

The differences in the metabolic patterns of bvFTD patients are in agreement with the results of a voxel-based data-driven study performed on a large sample of frontal variant FTD [45]. The authors showed different clustering of patients according to the hypometabolic patterns. While a first metabolic cluster included most of the lateral and medial prefrontal cortex, bilaterally, and correlated with performances on memory and executive neuropsychological tasks, other two clusters (left- and right-sided) comprised limbic regions such as the sub-callosal medial frontal region, the temporal pole, medial temporal structures and the striatum. In the present series, the metabolic differences could not be correlated to specific patterns of cognition at the single subject level. This may be due to the small sample size, and/or to the need for a more in-depth assessment of the multiple facets of executive function and social cognition abilities.

Overall, the heterogeneity of metabolic pattern may be related to the presence of different neuropathological substrates (e.g., frontotemporal lobar generation due to Tau or TDP-43 pathology). Despite the poor insight on the neurobiology underlying region-specific dysfunctions and behavioral symptoms in bvFTD, some evidence supports the idea of selective pathology-driven network vulnerability in course of this disease. In this context, a recent study in a murine model of FTD proved a selective vulnerability in salience network regions (i.e., ventral striatum and insula) that was directly associated with related behavioral abnormalities and higher levels of phosphorylated tau [46].

The integrity of salience network is crucial for the elaboration of social cues in a specific context, as proved by our correlation analysis results in bvFTD patients. Notably, the damage of the right amygdala was the main responsible of emotion recognition and attribution deficits. This brain structure plays a central role in the recognition of the affective valence of facial expressions, mostly of negative emotions, and it has been previously reported as a main correlate of poor facial and dynamic emotion recognition in bvFTD [8]. Reduced affective processing in bvFTD patients was also correlated to a significant hypometabolism in the temporal pole and anterior/middle cingulate cortex. As shown by functional MRI studies in normal individuals, both basic (pleasant or unpleasant) and social emotions (e.g., embarrassment or admiration), and physical or emotional pain significantly activate these structures, supporting them as key nodes for the system devoted to the interoceptive awareness of emotional states [42]. Moreover, these regions that appeared to be specifically dysfunctional in single bvFTD patients have a central role in the brain network dedicated to the empathic response [3]. Namely, while the insula is considered the convergence point of the social context processing information network [47], matching external signals to internal states, the cingulate cortex is involved in preparing an appropriate response to such states and the temporal regions in sorting of the value of target-context associations.

Correlation analyses revealed also the association between the hypometabolism in the bilateral insula and the impairment in disgust recognition. As suggested by previous fMRI studies [3], the anterior insula is a key node within the distributed viscero-autonomic/social–emotional network and it is implicated in disgust responding together with fronto-opercular regions [3]. These FDG-PET correlations providing new functional *in vivo* evidence for the selective damage of the insula in bvFTD are in agreement with the previously reported association between insular structural damage and difficulties to access internal sensations providing disgust cues at autonomic level [48].

Notwithstanding the lack of neuropathological confirmation, which is certainly a limit of the present study, hereby we described a group of patients with diagnosis of probable bvFTD. In particular, all patients presented with imaging biomarkers supportive for bvFTD and no subject had CSF values or FDG-PET metabolic patterns at the single-subject level suggestive for AD condition. Severe deficits of emotion recognition and processing emerged at the neuropsychological testing in the whole bvFTD group, according to what stated in the consensus criteria [24]. FDG-PET single-subject maps showed different patterns of hypometabolism at the individual level with a very consistent involvement of frontal-temporal and limbic regions, in line with the previous hypothesis- and data-driven findings [25, 45, 49–50]. Unfortunately, the small sample size prevents us to further investigate the apparent heterogeneity in performances at the social cognition tasks. Studies combining behavioral paradigms and imaging data are thus crucial to better clarify specific functional neural underpinnings of social cognition impairments in this neurodegenerative condition.

Finally, the addition of socio-emotional processing tasks within the standard neuropsychological assessment of bvFTD patients may provide an extra value for the early and differential diagnosis. Both our data and previous findings strongly suggest the use of high-order social information processing tasks on clinical setting. Although objective impairments on social cognition tasks do not yet represent a key criterion for the diagnosis of bvFTD, the “complaining of loss of empathy or sympathy” represents indeed a core clinical feature [24].
